# Matching-Updating Mechanism: A Solution for the Stable Marriage Problem with Dynamic Preferences

**DOI:** 10.3390/e24020263

**Published:** 2022-02-11

**Authors:** Akhmad Alimudin, Yoshiteru Ishida

**Affiliations:** 1 Department of Computer Science and Engineering, Toyohashi University of Technology, Toyohashi 441-8580, Japan; ishida@cs.tut.ac.jp; 2 Department of Multimedia Creative Technology, Politeknik Elektronika Negeri Surabaya, Surabaya 60111, Indonesia

**Keywords:** stable marriage problem, update matching, dynamic preferences

## Abstract

We studied the stable marriage problem with dynamic preferences. The dynamic preference model allows the agent to change its preferences at any time, which may cause instability in a matching. However, preference changing in SMP instances does not necessarily break all pairs of an existing match. Sometimes, only a few couples want to change their partners, while others choose to stay with their current partners; this motivates us to find stable matching on a new instance by updating an existing match rather than restarting the matching process from scratch. By using the update mechanism, we try to minimize the revision cost when rematching occurs. The challenge when updating a matching is that a cyclic process may exist, and stable matching is never achieved. Our proposed mechanism can update a match and avoid the cyclic process.

## 1. Introduction

Numerous types of research have been conducted on the stable matching problem in fields including computer science, mathematics, and economics. The term “matching” refers to a collection of agents wishing to form a pair that meets each agent’s criteria. The stable marriage problem (SMP) was introduced by Gale and Shapley [1]. It is one of the most well-known stable matching problems. Since its original conception in 1962, the SMP has attracted significant attention from researchers. Numerous extended variants of SMP have also emerged, such as the stable roommate problem, the college admissions problem, the hospital/resident problem, and several other stable matching problems [2,3,4,5]. The SMP algorithm has been widely used to solve several real-world problems. One of the most widely used variants is the hospital/resident problem variant [4,6], which is used to place medical students in hospitals or to select new students. Nowadays, the SMP algorithm is also widely applied to large-scale computer applications, such as content delivery networks [7] and job scheduling of virtual machines to servers [8,9].

The SMP is a bipartite matching problem with an equal number of agents on each side. Each agent expresses a strict order preference that includes all members of the opposite side. A matching μ is unstable when at least one blocking pair exists. A blocking pair is formed of a man *m* and a woman *w* who are not partners in a matching μ, but prefer each other over their current partners. In the classical SMP, each agent expresses a strict order preference for the opposite side. However, in real-world situations, some agents are occasionally unable to express their actual preference list due to a lack of information or observations about the opposing side, leading to the agents’ preferences changing dynamically. For example, consider job scheduling for virtual machines (VMs) to servers. Computer resource consumption is dynamic, and users must understand the behavior of their virtual machines to define the minimum and maximum resource requirements before deploying to the server. However, several users do not have the ability to define the minimum and maximum resource requirements for their virtual machines. If the assigned resources exceed the actual usage of their VM, this leads to a large amount of unused resources on the server. Meanwhile, the VM’s performance suffers if the allocated resources are insufficient. All virtual machines and servers need to be rescheduled to improve their effectiveness. When rescheduling, the scheduler determines the new preference for VMs and servers by examining the VMs’ historical information. This study aims to find a stable matching for the SMP with the dynamic preference model, where dynamic preferences refer to a scenario in which agents’ preferences might change dynamically over time. To illustrate the problem, we use a simple 3 × 3 SMP.

**Figure 1:**
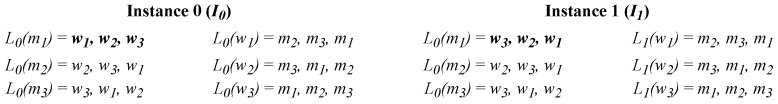
The 3 × 3 SMP with dynamic preferences.

**Example** **1.** *There is a set of men M = {$m_{1},m_{2},m_{3}$} and a set of women W = {$w_{1},w_{2},w_{3}$}. Assume that agent $m_{1}$ has dynamic preferences by changing his preference order. Specifically, these dynamic preferences generate two SMP instances, as depicted in Figure 1:*

Traditionally, we can use the Gale–Shapley algorithm to find a stable matching solution for each instance. In Example 1, we can find the stable matching for Instances 0 and 1 by processing it separately. However, a change in preference for an agent does not necessarily affect the stability of all pairs in a matching. Some small preference changes may not affect all agents, and only a few pairs want to change their partners, while others prefer to stay with their current partners. This motivates us to maintain stability in the SMP with dynamic preferences by using a matching-updating mechanism. The SMP with dynamic preferences is an extended version of the original SMP; the goal is to obtain a stable matching on the SMP instance. The Gale–Shapley algorithm is one way to find a stable matching for the SMP; by starting with an empty matching, the Gale–Shapley algorithm takes $O(n^{2})$ to find a stable matching. This study does not focus on the computational costs of finding a stable matching, but we try to minimize the revision costs when preferences change. The definition of revision cost here is the cost/impact when we rematch the instance. Using the Gale–Shapley algorithm, we can always find a stable matching, but the rematching process starts from the beginning (empty matching). We try to minimize the revision cost when rematching by using the match-updating mechanism. As a practical example, when we perform rematching between VMs and servers, if we perform the rematching using the Gale–Shapley algorithm, we need to involve all agents in the instance, which means all VMs and servers need to temporarily stop. However, we can minimize the number of VMs and servers that have to stop with the match-updating mechanism.

Our contribution in this research is the minimization of the revision cost when performing the rematching. Our theorems and mechanism demonstrate the process of finding stable matching by updating the previous matching. Our paper is organized as follows. Section 2 contains a number of studies relevant to this research. In Section 3, we discuss how to deal with dynamic preferences in the stable marriage problem by updating the previous stable matching. In Section 4, we discuss our findings and their impacts on future research. In Section 5, we provide the conclusions of our study.

## 2. Preliminaries

In this study, we deal with SMP instances that have dynamic preferences. In the dynamic preference model, the agent is allowed to change their preferences. The agent’s preference changes may affect the original SMP instance. In other words, changing the preference of one agent will create another SMP instance. Numerous studies on dynamic preferences in stable matching have been published. Kanade et al. [10] proposed an algorithm that maintains a matching for evolving preferences of stable matching. Evolving preference is similar to our dynamic preference model. The goal of their study was to maintain an approximately stable matching, whereas our goal is to find the stable matching for all available instances. Chen et al. [11] proposed the concept of robustness and the near-stability of matching for dynamic preferences. Tobustness means maintaining all instances produced by dynamic preferences that remain stable in a classical way, whereas near-stability tries to find the stability by measuring the strength of incentive between two agents in a blocking pair.

The aim of this study is to find a stable matching within dynamic instances by updating a previous stable matching. With respect to the creation of a new instance, the previous stable matching may be stable or unstable for the newly created instance. In order to update a stable matching, we need to identify the presence of a blocking pair in the matching. If no blocking pair is confirmed, it means that the given matching from the previous instance remains stable in the newly created instance. However, when a blocking pair is encountered in the matching, we need to satisfy the blocking pair. Otherwise, the matching will remain unstable. When updating a matching, it is essential to keep in mind the domino effect while we attempt to satisfy the blocking pair. The domino effect of attempting to satisfy the blocking pair means that we also need to break another pair. Furthermore, we must be able to re-satisfy the pair that was previously disengaged by the previous step. When we attempt to revise a pair in matching, the worst-case scenario is that an infinite loop occurs in a process. As in the open question of Knuth [12], which states that there is a cyclic process when attempting to satisfy the blocking pair using the Gale–Shapley algorithm, this situation leads to the matching remaining unstable. Knuth’s open question also shows that updating a stable matching using the Gale–Shapley algorithm is not possible.

Roth and Vande [13] demonstrated in their work that stable matching is always possible, even when starting with arbitrary matching. Additionally, this research addressed Knuth’s [12] open question. Jinpeng Ma [14] examined the Roth and Vande (RV) mechanism and admitted that stable matching will always be found with a probability of one when using the RV mechanism, even though it starts from arbitrary matching. Unlike the Gale–Shapley algorithm, which always produces the same stable matching, the RV mechanism could produce a variety of stable matchings for each execution, including man-optimal, woman-optimal, and some fair matching (intermediate) in the lattice structure. However, Jinpeng Ma also claimed that in the RV mechanism, not all stable matching options can be achieved using the RV mechanism. In our study, we try to update the stable matching by modifying the Roth and Vande mechanism. In our dynamic preference model, we have the previous SMP instance and its stable matching. Furthermore, we use those properties as input for our proposed algorithm.

### 2.1. The Stable Marriage Problem

The stable marriage problem (SMP) was introduced by Gale and Shapley [1]. An SMP is a two-sided matching problem with an equal number of agents on each side. Each agent expresses a strict order preference that includes all members of the opposite side. The goal of the Gale–Shapley algorithm is to find a stable matching for all involved agents. The term “matching” refers to the process of pairing or matching participants (groups of men and women) in order to satisfy a specified criterion. A matching process is a step to determine the pairs of participants (sets of men and women) to meet the specified criteria. The size *n* of the SMP instance is I=(M,W,L), where *M* denotes the set of male agents $M=\{m_{1},m_{2},\ldots ,m_{n}\}$, *W* denotes the set of female agents $W=\{w_{1},w_{2},\ldots ,w_{n}\}$, and *L* represents a list of the preference order of an agent for the opposite sex. To express the preference order list of agent $m_{1}$, we can denote it by $L(m_{1})$. For such an instance, a matching μ is a one–one correspondence between the men and the women. If a man *m* and a woman *w* are matched in μ, they are referred to as partners, and we write m=μ(w) and w=μ(m). μ(m) is the partner of *w* in μ, and μ(w) is the partner of *m* in μ. In the SMP, the stability of matching is determined by the existence of a blocking pair. In a matching μ, a pair (m,w) is said to be a blocking pair if *m* and *w* are not a pair in μ, but *m* prefers *w* over μ(m) and *w* prefers *m* over μ(w). That is, $\left(w{≻}_{m}\mu \left(m\right)\right)\wedge \left(m{≻}_{w}\mu \left(w\right)\right)$. A matching that contains at least one blocking pair is called unstable; otherwise, it is stable.

### 2.2. Dynamic Preferences in the SMP

**Figure 2:**
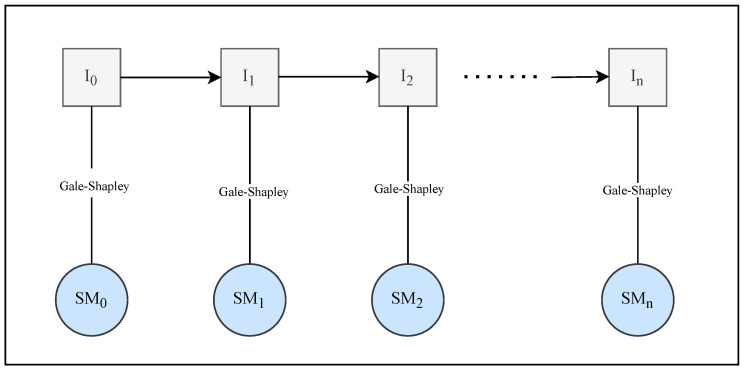
Illustration of solving every instance of the SMP using the Gale–Shapley algorithm.

In the traditional SMP, each agent’s preference order or rank is fixed. As discussed in the introduction, we attempted to solve the SMP with a dynamic preference model. In this study, we assume that the preference list of agents is complete, meaning that each agent must include all available members of the opposite side in their preference list. As illustrated in Figure 2, a preference change of agents in the SMP instance may lead to the creation of another SMP instance.

**Definition** **1.** *The dynamic preferences describe a situation in which an agent’s preference for an SMP instance can change through permutation at anytime, leading to the formation of another SMP instance.*

As a consequence of the preference changes, a new instance will be created based on the number of preferences expressed by agents.

**Definition** **2.** *A dynamic instance is a set of SMP instances generated from an original SMP instance with dynamic preferences.*

Dynamic preferences result in a more realistic scenario for a stable matching problem in which agents do not always express their preferences correctly due to a lack of observations or information about their opposite side. In the classical SMP, an instance is defined as I=(M,W,L), where *M* and *W* denote the sets of male and female agents, respectively; *L* denotes the order of each agent’s preference list for the opposite side. The preference list of a male agent *m* can be written as *L*(*m*). However, in the case of the SMP with dynamic preferences, multiple SMP instances may exist as a result of changes in one or more agents’ preferences. In other words, the number of possible instances that will occur is $i=k_{1}\times k_{2}\times \cdots \times k_{2n}$, where *k* denotes the number of preferences expressed by each agent, and *n* is the size of the SMP. The dynamic instance set is $DI=\{I_{1},I_{1},\cdots ,I_{i}\}$. For example, if there is a single agent with two distinct preferences in size *n* of the SMP, there will be two SMP instances available. In the dynamic preference model, changes in an agent’s preferences are a permutation of their previous preference list. This means that no new members will be added or removed from an agent’s preference list during the preference changes.

## 3. Updating the Stable Matching

**Figure 3:**
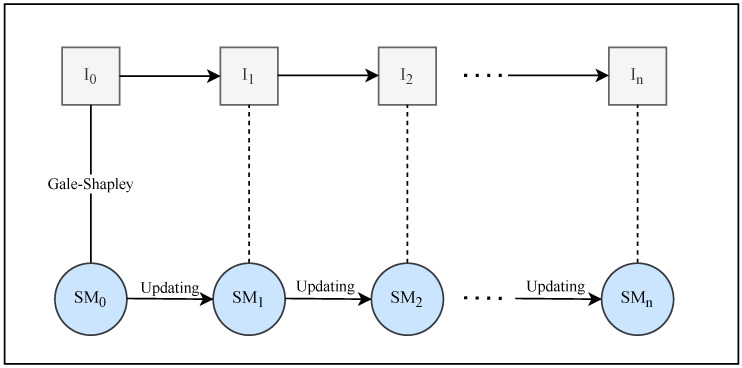
Illustration of finding the stable matching of the SMP by updating the previous matching.

As mentioned in the introduction, our goal is to find a stable matching for each instance. This study attempts to find stable matching for each instance by updating the previous instance’s stable matching. The steps of finding stable matching using the previous matching update mechanism are depicted in Figure 3.

Our steps for determining stable matching for a dynamic instance are as follows:

1. Determining the impacts of modifying an agent’s preferences: We identify the effects of changing an agent’s preference and determine whether it leads to the occurrence of a blocking pair in a matching.
2. Initiating an update of the matching if a blocking pair exists.
3. If there is no blocking pair, the previous instance’s matching will likely be stable for the new instance.

### 3.1. Identifying the Preference Changes

The preference change of an agent does not always affect the stability of a previous matching. We classify the types of changes in an agent’s preferences as either major or minor changes.

**Definition** **3.** *A minor preference change is a permutation-based change in an agent’s preference that does not affect the pair in a stable matching. As a result, this preference change has no direct impact on the creation of blocking pairs.*

**Definition** **4.** *A major preference change is a permutation-based change in an agent’s preference that leads to the creation of a blocking pair. These preference changes may result in the instability of a matching.*

Every agent is allowed to change their preferences at any time in the dynamic preference model. A change in an agent’s preference does not always lead to a change in matching. However, it can occasionally allow for the appearance of blocking pairs, leading to the instability of a previous matching.

Given an SMP with dynamic preferences of size *n* and with the initial instance $I_{0}=\left(M_{0},W_{0},L_{0}\right)$ and stable matching $\mu_{0}$, where $M_{0}=\left\{m_{1},m_{2},\ldots ,m_{n}\right\},W_{0}=\left\{w_{1},w_{2},\ldots ,w_{n}\right\}$, if we suppose that a male agent ($m_{1}$) changes his preference through permutation, we will have another instance of the SMP: $I_{1}=\left(M_{1},W_{1},L_{1}\right)$, where $L_{0}\left(m_{1}\right)\neq L_{1}\left(m_{1}\right)$. By referencing the previous SMP instance, we determine the occurrence of potential blocking pairs. We identify whether the preference change for agent $m_{1}$ is a minor change or not.

**Theorem** **1.** *Under the assumption that the preference change occurs on only one side, and if no agent with a rank lower than $\mu_{0}\left(m_{1}\right)$ in $L_{0}\left(m_{1}\right)$ becomes higher than $\mu_{0}\left(m_{1}\right)$ in $L_{1}\left(m_{1}\right)$, the change is considered minor.*

**Proof.**  Assume that the position of agent $\mu_{0}\left(m_{1}\right)$ in $L_{0}\left(m_{1}\right)$ is the ${\left(n-1\right)}^{th}$ rank; this means that agents from the first rank to the ${\left(n-2\right)}^{th}$ rank in $L_{0}\left(m_{1}\right)$ prefer other male agents over $m_{1}$. The agent at the $n^{th}$ rank is a worse agent than $\mu_{0}\left(m_{1}\right)$, which means that $\mu_{0}\left(m_{1}\right)$ is the best option for $m_{1}$ at $I_{0}$ (man-optimal). Thus, as long as the $n^{th}$ agent’s position remains lower than $\mu_{0}\left(m_{1}\right)$ in $L_{1}\left(m_{1}\right)$, any permutation in the agent from the 1^*st*^ rank to $\mu_{0}\left(m_{1}\right)$ in $L_{0}\left(m_{1}\right)$ does not affect the pair ($m_{1}$, $\mu_{0}\left(m_{1}\right)$). This results in $\mu_{0}\left(m_{1}\right)$ = $\mu_{1}\left(m_{1}\right)$.    □

Based on Theorem 1, we describe the following corollaries.

**Corollary** **1.** *If $\mu_{0}\left(m_{1}\right)$ is the last choice of $m_{1}$ in $L_{0}\left(m_{1}\right)$, then any permutation in $L_{0}\left(m_{1}\right)$ leads to minor change.*

**Proof.**  If $\mu_{0}\left(m_{1}\right)$ is the last order in $L_{0}\left(m_{1}\right)$, $\mu_{0}\left(m_{1}\right)$ is considered the best choice that $m_{1}$ can get because other female agents prefer other male agents over $m_{1}$. As a result, ($m_{1}$, $\mu_{0}\left(m_{1}\right)$) will not change, although the rank of $\mu_{0}\left(m_{1}\right)$ increases in $L_{1}\left(m_{1}\right)$.    □

**Corollary** **2.** *If $\mu_{0}\left(m_{1}\right)$ becomes the first choice of $m_{1}$ in $L_{1}\left(m_{1}\right)$, then it is a minor change.*

**Proof.**  If $\mu_{0}\left(m_{1}\right)$ is the first option in $L_{1}\left(m_{1}\right)$, then no agent that is worse than $\mu_{0}\left(m_{1}\right)$ in $L_{0}\left(m_{1}\right)$ becomes better than $\mu_{0}\left(m_{1}\right)$ in $L_{1}\left(m_{1}\right)$. Moreover, if the rank of $\mu_{0}\left(m_{1}\right)$ increases in $L_{1}\left(m_{1}\right)$, it strengthens the pair between $\mu_{0}\left(m_{1}\right)$ and $m_{1}$.    □

**Corollary** **3.** *If there is any permutation in $L_{0}\left(m_{1}\right)$ from the first rank to $\mu_{0}\left(m_{1}\right)$ or any permutation from the rank of $\mu_{0}\left(m_{1}\right)$ + 1 to the $n^{th}$ rank, then it is a minor change.*

**Proof.**  The permutation of the first rank to $\mu_{0}\left(m_{1}\right)$ does not lead to a worse agent being superior to $\mu_{0}\left(m_{1}\right)$. Meanwhile, permutations from the rank of $\mu_{0}\left(m_{1}\right)$ +1 to the lowest-ranked agent will also be inferior to $\mu_{0}\left(m_{1}\right)$. According to Theorem 1, it is a minor change if no agent worse than $\mu_{0}\left(m_{1}\right)$ at $L_{0}\left(m_{1}\right)$ improves in $L_{1}\left(m_{1}\right)$.    □

Theorem 1 and its corollary define the constraints under which the effect of changing agents on male agents (proposing side) can be identified. However, Theorem 1 and its corollary hold true when female agents (the proposed side) possess a change in their preferences. We identify the impact of changing an agent’s preferences using Theorem 1 and its corollary and whether the change has the potential to lead to the establishment of a blocking pair in the new instance or not. This identification accelerates the decision making regarding updating a matching.

We define Algorithm 1 to detect potential blocking pairs based on Theorem 1 and its corollaries. If Theorem 1 and its corollaries are satisfied in an agent, the agent might not have an incentive to change his/her partner. However, if Theorem 1 and its corollary are not satisfied, a deeper checking process using the stability-checking algorithm [3] will be performed. If a blocking pair is detected, the algorithm will mark the pair for removal and initiate the update of the matching process.

Theorem 1 assumes that the preference changes only occur on one side. Theorem 1 is used to determine the type of preference change that occurs in an agent, i.e., whether it is a major or minor change. The aim is to determine whether the preference changes incentivize an agent to change their partner. By checking the type of preference change for all agents with Theorem 1, we are still able to identify the potential blocking pair, even though both sides of the agents change their preferences simultaneously. Given an SMP with dynamic preferences of size *n* and with initial instance $I_{0}=\left(M_{0},W_{0},L_{0}\right)$ and stable matching *μ*_0_, where $M_{0}=\left\{m_{1},m_{2},\ldots ,m_{n}\right\},W_{0}=\left\{w_{1},w_{2},\ldots ,w_{n}\right\}$, if we suppose that a male agent ($m_{1}$) and his partner ($\mu (m_{1})$) change their preference simultaneously, we will have another instance of the SMP: $I_{1}=\left(M_{1},W_{1},L_{1}\right)$, where $L_{0}\left(m_{1}\right)\neq L_{1}\left(m_{1}\right)$ and $L_{0}\left(\mu \left(m_{1}\right)\right)\neq L_{1}\left(\mu \left(m_{1}\right)\right)$.
**Algorithm 1** Finding a potential blocking pair.**Input:** -Current SMP Instance: mPref, wPref -Previous SMP Instance: prevMPref, prevWPref, Stable Matching (SM)
   1:**for** m, w in SM **do**   2:    **if** mPref[m] != prevMPref[m] **then**   3:        **for** prevW in prevMPref[m] **do**   4:           **if** prevMPref[m].rank(w) > prevMPref[m].rank(SM(prevW)) **then**   5:                potentialBP.append(prevW)   6:           **end if**   7:        **end for**   8:        **for** woman in mPref[m] **do**   9:           **if** woman in potentialBP **then** 10:               **if** wPref[woman].rank(m) > wPref[woman].rank(SM(woman)) **then** 11:                   removedPair.append(m, w) 12:               **end if** 13:           **end if** 14:        **end for** 15:    **end if** 16:**end for**

**Corollary** **4.** *If $m_{1}$ and $m\mu_{0}\left(m_{1}\right)$ confirm a minor change in $I_{1}$, then the pair ($m_{1}$, $m\mu_{0}\left(m_{1}\right)$) does not trigger a blocking pair.*

**Proof.**  If agent $m_{1}$ confirms that his preference change is minor, $\mu_{0}\left(m_{1}\right)$ is the best possible woman that agent $m_{1}$ can get. In addition, if agent $\mu_{0}\left(m_{1}\right)$ also confirms that her preference change is minor, then agent $m_{1}$ is the best possible option for agent $\mu_{0}\left(m_{1}\right)$ because the proposer is $m_{1}$. Thus, if both agents agree that their current partners are the best partners they can get, then the preference changes of agents $\mu_{1}$ and $\mu_{0}\left(m_{1}\right)$ do not have the potential to create a blocking pair.    □

It is worth noting that Theorem 1 is employed to determine whether a change in an agent’s preference can motivate an agent to change their current partner. Theorem 1 is used to verify agents’ wishes about their partners following their preference changes. In other words, although both agents in a pair confirm a minor change, the pair is still likely to change if provoked by another agent that does not confirm a minor change.

### 3.2. Initiating the Matching Update

Once a blocking pair is confirmed to exist in a matching, the next step is to initiate a matching update. We modified Roth’s and Vande’s mechanism to update the stable matching. Random Paths to Stability [13] is a mechanism for discovering a stable matching solution by satisfying the matching’s blocking pair. Ref. [14] summarized the RV mechanism and admitted that stable matching can always be achieved with a probability of one starting from satisfying blocking pairs in arbitrary matching. Roth’s and Vande’s work can be analogized as follows:

1. Imagine that there is one room with one entrance; randomly select a pair from each matching process. Let the selected pair enter the room. The selected pair can be confirmed as a stable matching in this room because no other choice can break the pair. Meanwhile, the rest of the agents form a queue outside the room to enter the room one by one.
2. Ask an agent who is in front of the room to enter the room. There will be a matching process inside the room. The door of the room will remain closed before a stable matching is formed in the room.
3. Repeat the second step until there are no remaining queues and a stable matching is obtained. As a result, a stable matching will be obtained without any blocking pairs.

The RV mechanism begins the matching process by asking a single random pair to enter the room. Typically, a blocking pair will be selected in the initialization process. Following that, the remaining agents in the queue will be asked to enter the room separately. In the RV mechanism, new agents are not permitted to enter the room until all agents in the room reach stability. Therefore, this motivated us to shorten the finding of stable matching in the RV mechanism. The RV mechanism begins the matching process with a single pair for the initial part, while our proposed algorithm enables the initial process with multiple pairs. This significantly speeds up the process of finding stable matches by introducing multiple pairs into the room simultaneously. Here is our mechanism for updating a stable matching:

1. Imagine that there is one room with one entrance; select some pairs that have been confirmed as a stable matching. Let of all the selected pairs enter the room together. Meanwhile, the rest of the agents form a queue outside the room to enter the room one by one.
2. Ask an agent who is in front of the room to enter the room. There will be a matching process inside the room. The door of the room will remain closed before a stable matching is formed in the room.
3. Repeat the second step until there is no remaining queue and a stable matching is obtained. As a result, a stable matching will be obtained without any blocking pairs.

**Figure 4:**
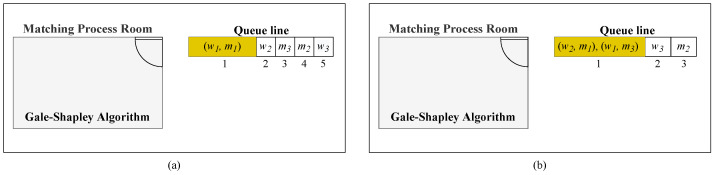
Comparison of the original Roth and Vande mechanism (**a**) and our update mechanism (**b**).

Figure 4 shows an illustration of the original RV mechanism and our updating mechanism. Our mechanism allows more than one pair to enter the room at the initial stage.

### 3.3. Reducing the Previous Matching

Given an SMP of size *n* with an instance I=(M,W,L), where M={$m_{1},m_{2},\ldots ,m_{n}$}, W={$w_{1},w_{2},\ldots ,w_{n}$}, the matching μ is unstable if there exists a blocking pair $\left(m_{i},w_{j}\right)$ such that $\left(w_{j}{≻}_{m_{i}}\mu \left(m_{i}\right)\right)\wedge \left(m_{i}{≻}_{w_{j}}\mu \left(w_{j}\right)\right)$ for $m_{i}\in M,w_{j}\in W$, where i=1,2,…,n and j=1,2,…,n. Equivalently, the stability for matching μ can be expressed as: $\left(\mu \left(m_{i}\right){≽}_{m_{i}}w_{j}\right)$∨$\left(\mu \left(w_{j}\right){≽}_{w_{j}}m_{i}\right)$ for $\forall m_{i}\in M,\forall w_{j}\in W$, where i=1,2,…,n and j=1,2,…,n.

**Theorem** **2.** *If we remove any one pair from the stable matching μ and SMP instance I, the reduced matching $\mu_{r}$ will remain stable for the reduced instance $I_{r}$.*

**Proof.**  Without loss of generality, we assume the removal of $\left(m_{1},w_{1}\right)=\left(m_{1},\mu \left(m_{1}\right)\right)$. Then, $M{\;}^{\prime }\equiv M\backslash m_{1}$ and $W{\;}^{\prime }\equiv W\backslash w_{1}$, $\mu_{r}=\left\{\left(m_{2},\mu \left(m_{2}\right)\right),\ldots ,\left(m_{n},\mu \left(m_{n}\right)\right)\right\}$. The stability condition for the matching $\mu_{r}$ is expressed as: $\left(\mu \left(m_{i}\right){≽}_{m_{i}}w_{j}\right)\vee \left(\mu \left(w_{j}\right){≽}_{w_{j}}m_{i}\right)$ for $\forall m_{i}\in M{\;}^{\prime },\forall w_{j}\in W{\;}^{\prime }$, where i=2,…,n and j=2,…,n.

When we remove $m_{1}$ from the preference list of women and $w_{1}$ from the preference list of men, there are only two possibilities for men: $m_{i}$ prefers $w_{1}$ to the partner $\mu (m_{i})$ in the stable matching μ, or $m_{i}$ prefers the partner $\mu (m_{i})$ to $w_{1}$ in the stable matching μ. Likewise, there are only two possibilities for women, which makes 2 × 2 = 4 combined cases altogether. We will check the stability condition above for the reduced matching $\mu_{r}$ for the reduced instance $I_{r}$.

**Case 1:** If $\left(w_{1}{≻}_{m_{i}}\mu \left(m_{i}\right)\right)$ and $\left(\mu \left(w_{j}\right){≻}_{w_{j}}\mu \left(m_{1}\right)\right)$, then the statement $\left(\mu_{r}\left(w_{j}\right){≽}_{w_{j}}m_{i}\right)$ for the stability condition of matching $\mu_{r}$ is true.

**Case 2:** If $\left(\mu \left(m_{i}\right){≻}_{m_{i}}w_{1}\right)$ and $\left(m_{1}{≻}_{w_{j}}\mu \left(w_{j}\right)\right)$, then the statement $\left(\mu_{r}\left(m_{i}\right){≽}_{m_{i}}w_{j}\right)$ for the stability condition of matching $\mu_{r}$ is true.

**Case 3:** If $\left(\mu \left(m_{i}\right){≻}_{m_{i}}w_{1}\right)$ and $\left(\mu \left(w_{j}\right){≻}_{w_{j}}\mu \left(m_{1}\right)\right)$, then both statements $\left(\mu_{r}\left(m_{i}\right){≽}_{m_{i}}w_{j}\right)$ and $\left(\mu_{r}\left(w_{j}\right){≽}_{w_{j}}m_{i}\right)$ for the stability conditions of matching $\mu_{r}$ are true.

**Case 4:** If $(w_{1}{≻}_{m_{i}}$ and $\left(m_{1}{≻}_{w_{j}}\mu \left(w_{j}\right)\right)$, since both conditions of agents cannot directly provide the true statement, then we need further investigation for this condition: $m_{i}$ and $w_{j}$ are a pair μ and are also in $\mu_{r}$. Then, we can write $m_{i}=\mu \left(w_{j}\right)$ and $w_{j}=\mu \left(m_{i}\right)$. If we remove $w_{1}$ from the men’s preferences, then $\mu (m_{i})$ increases; still, we cannot confirm whether $\mu (m_{i})$ becomes $m_{i}$’s first choice or not. If we assume that $\mu (m_{i})$ is not $m_{i}$’s first choice, then $m_{i}$ prefers another woman $\left(w_{x}\right)$ over $\mu (m_{i})$, that is, $\left(w_{x}{≻}_{m_{i}}\mu \left(m_{i}\right)\right)$. Currently, the partner of $w_{x}$ is $\mu (w_{x})$. Since μ is stable, then $\left(m_{i}{≻}_{w_{x}}\mu \left(w_{x}\right)\right)$ is not possible in μ, although we also remove $m_{1}$ from the women’s preferences. Then, $w_{x}$ and $m_{i}$ never become partners in μ. Thus, the statement $\left(\mu_{r}\left(m_{i}\right){≽}_{m_{i}}w_{j}\right)$ holds true for the stability condition of matching $\mu_{r}$. The true condition holds for all possible combinations of pair removals. That said, the stability condition for matching $\mu_{r}$ still holds true.    □

As discussed in Section 3.2, we meant to obtain as many pairs as possible for the initial stage. The pairs wishing to enter at the initial step must be stable with each other. Therefore, we reduce the size of the previous matching to obtain a stable matching with a smaller size. The reduction procedure is performed by removing the unwanted pairs (*m*, *w*) from a matching μ that causes the formation of a blocking pair. It should be noted that removing *m* and *w* from the previous matching is not permanent. The removal is intended to keep the remaining pairs stable. Thus, the stable reduced matching can be used as the initial stage for finding a stable matching. To accomplish this, we must introduce a procedure for determining which pairs to exclude from the matching μ. By using Theorem 2, we can safely remove any pair from the previous matching to get the reduced stable matching.

**Figure 5:**
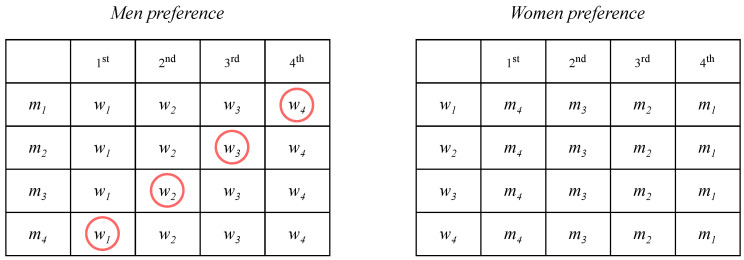
A 4 × 4 SMP instance before reduction.

Consider the following examples in Figure 5.

**Figure 6:**
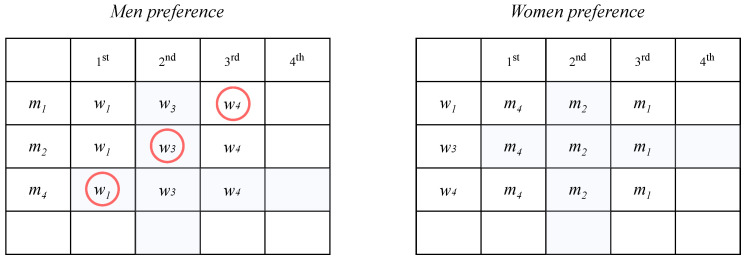
A 4 × 4 SMP instance before reduction.

We have stable matching (man-optimal) {$\left(w_{1},m_{4}\right),\left(w_{2},m_{3}\right),\left(w_{3},m_{2}\right),\left(w_{4},m_{1}\right)$}. Now, we will try to reduce the instance by randomly removing one pair in the stable matching. Suppose that we remove ($w_{2},m_{3}$). Thus, the size of the SMP instance will change to 3 × 3, as depicted in Figure 6.

Figure 6 shows the result of reducing the SMP instance from 4 × 4 to 3 × 3 by randomly deleting one pair, which keeps the reduced matching stable with respect to the reduced instance.

**Figure 7:**
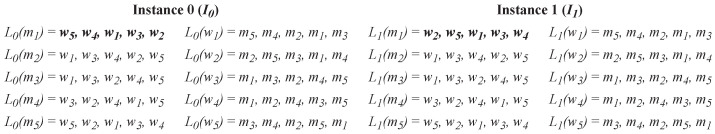
The 5 × 5 SMP instances with dynamic preferences.

**Example** **2.** *Given the SMP instance I = (M, W, L), a set of men M = $\left\{m_{1},m_{2},m_{3},m_{4},m_{5}\right\}$, and a set of women W = $\left\{w_{1},w_{2},w_{3},w_{4},w_{5}\right\}$, we assume that agent $m_{1}$ changes his preference and generate two SMP instances, as depicted in Figure 7:*

The stable matching of instance 0 is $\mu_{0}$ = {$\left(w_{1},m_{2}\right),\left(w_{2},m_{4}\right),\left(w_{3},m_{3}\right),\left(w_{4},m_{1}\right),\left(w_{5},m_{5}\right)$}. Now, we want to find the stable matching of instance 1 by updating $\mu_{0}$. Comparing $L_{0}$ and $L_{1}$, it is known that the pair $\left(w_{3},m_{1}\right)$ will become a blocking pair in matching $\mu_{0}$ if we use $L_{1}$ as the preference. $\left(w_{3},m_{1}\right)$ blocks $\left(w_{3},m_{3}\right)$ and $\left(w_{4},m_{1}\right)$ in matching $\mu_{0}$. By using Theorem 2, we allow the removal of the pair $\left(w_{4},m_{1}\right)$. Removing $\left(w_{4},m_{1}\right)$ from instance 1 will produce the reduced matching $\mu_{r}$ = {$\left(w_{1},m_{2}\right),\left(w_{2},m_{4}\right),\left(w_{3},m_{3}\right),\left(w_{5},m_{5}\right)$}, which is stable with respect to the reduced SMP instance ($I_{r}$). As illustrated in Figure 4b, members of $\mu_{r}$ may immediately enter the room together. Meanwhile, the removed pair $\left(w_{4},m_{1}\right)$ forms a queue outside the room.

Algorithm 2 summarizes our work in this study. By combining Algorithm 1 with the reduced pair, we can start updating the previous matching and obtain the new stable matching for a new instance. Since we can start the initial stage by processing several pairs simultaneously, the revision cost of rematching can be minimized.

### 3.4. Controlling the Matching Orientation

An exciting aspect of the RV mechanism is the variety of the stable matchings obtained. Unlike the Gale–Shapley algorithm, which always provides the same stable matching, the RV mechanism can produce a different stable matching for each execution. While it is impossible to guarantee that all stable matchings in the lattice structure are obtained, man- and woman-optimal matching can be guaranteed. According to Jinpeng Ma’s research, certain circumstances can result in a stable match that leads to a particular orientation or, at the very least, in close proximity to the optimal orientation within the lattice structure. According to his study, if the last agent in the matching process is a male agent, a man-optimal or fairly stable match is obtained that is close to the man-optimal orientation in the lattice structure. In the opposite direction, if the last agent in the matching process is a woman, it will obtain a woman-optimal or fair stable matching that is close to the woman-optimal orientation in the lattice structure. This allows us to control the orientation of the stable matching that we want to produce by sorting the *queueMember* variable in Algorithm 2. By simply putting all male agents at the end of the queue, we will be able to obtain the man-optimal matching, or we can put all the female agents at the end of the queue if we want woman-optimal matching.
**Algorithm 2** Updating stable matching.**Input:** -Current SMP Instance: mPref, wPref -Previous SMP Instance: prevMPref, prevWPref, Stable Matching (SM)
   1:**for** m, w in SM **do**   2:    **if** mPref[m] != prevMPref[m] **then**   3:        **for** prevW in prevMPref[m] **do**   4:           **if** prevMPref[m].rank(w) > prevMPref[m].rank(SM(prevW)) **then**   5:                potentialBP.append(prevW)   6:           **end if**   7:        **end for**   8:        **for** woman in mPref[m] **do**   9:           **if** woman in potentialBP **then** 10:               **if** wPref[woman].rank(m) > wPref[woman].rank(SM(woman)) **then** 11:                   removedPair.append(m, w) 12:               **end if** 13:           **end if** 14:        **end for** 15:    **end if** 16:    **if** wPref[w] != prevWPref[w] **then** 17:        **for** prevM in prevWPref[w] **do** 18:           **if** prevWPref[w].rank(m) > prevWPref[w].rank(SM(prevM)) **then** 19:                potentialBP.append(prevM) 20:           **end if** 21:        **end for** 22:        **for** man in wPref[w] **do** 23:           **if** man in potentialBP **then** 24:               **if** mPref[man].rank(w) > mPref[man].rank(SM(man)) **then** 25:                   removedPair.append(m, w) 26:               **end if** 27:           **end if** 28:        **end for** 29:    **end if** 30:**end for** 31:SM = SM - removedPair 32:roomMember = member of SM 33:queueMember = member of removedPair 34:**for** newMember in queueMember **do** 35:    roomMember.append(newMember) 36:    /* see the Appendix A Algorithm A1 for pathToStability function */ 37:    pathToStability(newMember) 38:**end for**

## 4. Discussion

We propose an algorithm for finding stable matching in the SMP with dynamic preferences. In the SMP with dynamic preferences, an agent’s preferences might change dynamically, affecting the stability of the previous stable matching. One traditional method for maintaining matching stability is to perform rematching each time the preferences change, as illustrated in Figure 2. However, in some situations, preference changes do not always affect the matching stability, such as with minor changes in preference. In other words, the prior matching remains stable in the presence of the new preference. This motivates us to update the matching to find a stable matching with dynamic preferences. In certain circumstances, such as when the preferences changes are minor, the match-updating method can accelerate the discovery of a stable match for the new instance. In this study, we are not focused on the computational cost while performing the rematching process. With the match-updating mechanism, we try to minimize the revision cost that needs to be paid.

Our current study is a preliminary step toward resolving issues in the SMP with dynamic preferences. In the SMP with the dynamic preferences, changing an agent’s preferences will generate new instances, which may affect the stability of the matching obtained. Refs. [15,16] investigated the new concept of stability for stable matching problems with dynamic preferences. They tried to broaden the definition of stability in stable matching problems with dynamic preferences. The concept of new stability in stable matching problems with dynamic preferences was determined by measuring the strength of each founded matching against all available instances. To measure a matching’s stability and strength, they needed to find a stable matching for each instance. Our current study will positively impact this problem by empowering the discovery of stable matching for each available stable matching instance. Our findings will aid future research in stable matching, especially with the dynamic preference model. The process of determining stable matching in an SMP with dynamic preferences can be accelerated by employing our theorem and proposed algorithm.

In our previous studies [8,9], we tried to find a matching between containers and servers. A matching process was carried out by using the Gale–Shapley algorithm between three containers and three servers [9]. The rematching process was performed in the traditional way by processing the new preferences using the Gale–Shapley algorithm. In our previous study, we also tried to find the matching between containers and servers under probabilistic preferences [8]. We focused on managing the rematching process between containers and servers when dealing with dynamic preferences. When an agent’s preferences changed, the container scheduler performed the rematching process. With our current research, we can speed up rematching by updating the previous matching. Our match-updating method can accelerate the search for a stable matching for the new instance if the preference changes are minor. However, this update method still has limitations and weaknesses. For some situations, such as if all agents’ preferences are changed and no one confirms the minor preference changes, the match-updating process cannot be accelerated. Moreover, if all agents want to change their current partners, the computational cost of finding a stable matching in a new instance will be higher than with the traditional way of finding a stable matching due to the extra cost of the preference change checking process.

## 5. Conclusions

In the SMP with dynamic preferences, agents can modify their preferences at any time, leading to the creation of a new SMP instance. As the consequence of the preference changes, the matching obtained may become unstable. We proposed a mechanism for finding a stable matching by updating an existing matching. A cyclic process (infinite loop) may exist when updating a matching. We employed the RV mechanism to prevent this cyclic process when updating a matching. We attempted to update a matching by isolating the pairs that contribute to its instability, thus generating a smaller matching with a stable condition (reduced stable matching). Our proposed mechanism can update a matching without worrying about the cyclic process. As a result, our match-updating method can accelerate the process of finding a stable matching for a new instance when the preference changes are minor. That said, the main contributions of this paper are Theorems 1 and 2, which can shorten the search for stable matching with dynamic preferences. Thus, we can minimize the revision cost when performing the rematching.

For the SMP with dynamic preferences, our current work is a preliminary step toward resolving the bigger issues of the SMP with dynamic preferences. This paper aims to find a stable matching for each generated instance of the SMP with dynamic preferences. However, defining the new stability concept for the SMP with dynamic preferences would be the interesting part of the SMP with dynamic preferences.

## Data Availability

Not applicable.

## Appendix A

**Algorithm A1** Procedure of the path to stability.
**Input:** -SMP Instance (manPrefers and womanPrefers) 1: M = {} 2: **procedure**PathToStability(newMember) 3: freeMember.append(newMember) 4: **while** freeMember **do** 5: proposer = freemember.pop(0) 6: **if** proposer == woman **then** 7: womanList = womanPrefers[proposer] 8: **for** man in womanList **do** 9: **if** man is roomMember **then** 10: manList = manPrefers[man] 11: **if** man in M **then** 12: manFiance = M(man) 13: **if** manList.index(proposer) < manList.index(manFiance) **then** 14: freeMember.append(manFiance) 15: M(proposer) = man 16: **end if** 17: **else** 18: M(proposer) = man 19: **end if** 20: **end if** 21: **end for** 22: **else** 23: manList = manPrefers[proposer] 24: **for** woman in manList **do** 25: **if** woman is roomMember **then** 26: wList = womanPrefers[woman] 27: **if** woman in M **then** 28: womanFiance = M(woman) 29: **if** wList.index(proposer) < wList.index(womanFiance) **then** 30: freeMember.append(womanFiance) 31: M(proposer) = woman 32: **end if** 33: **else** 34: M(proposer) = woman 35: **end if** 36: **end if** 37: **end for** 38: **end if** 39: **end while** 40: return M 41: **end procedure**

## References

[1] Gale D., Shapley L.S. (1962). College admissions and the stability of marriage. Am. Math. Mon..

[2] Irving R.W. (1985). An efficient algorithm for the “stable roommates” problem. J. Algorithms.

[3] Gusfield D., Irving R.W. (1989). The Stable Marriage Problem: Structure and Algorithms.

[4] Manlove D.F. (2008). Hospitals/residents problem. Encyclopedia of Algorithms.

[5] Iwama K., Miyazaki S. A survey of the stable marriage problem and its variants. Proceedings of the International Conference on Informatics Education and Research for Knowledge-Circulating Society (ICKS 2008).

[6] Irving R.W., Manlove D.F., Scott S. (2000). The hospitals/residents problem with ties. Scandinavian Workshop on Algorithm Theory.

[7] Dilley J., Maggs B., Parikh J., Prokop H., Sitaraman R., Weihl B. (2002). Globally distributed content delivery. IEEE Internet Comput..

[8] Alimudin A., Ishida Y. (2020). Dynamic assignment based on a probabilistic matching: Application to server-container assignment. Procedia Comput. Sci..

[9] Alimudin A., Ishida Y. Service-Based Container Deployment on Kubernetes Using Stable Marriage Problem. Proceedings of the 2020 the 6th International Conference on Frontiers of Educational Technologies.

[10] Kanade V., Leonardos N., Magniez F. (2015). Stable matching with evolving preferences. arXiv.

[11] Chen J., Skowron P., Sorge M. (2021). Matchings under preferences: Strength of stability and tradeoffs. ACM Trans. Econ. Comput..

[12] Knuth D.E. (1997). Stable Marriage and Its Relation to Other Combinatorial Problems: An Introduction to the Mathematical Analysis of Algorithms.

[13] Roth A.E., Vate J.H.V. (1990). Random paths to stability in two-sided matching. Econom. J. Econom. Soc..

[14] Ma J. (1996). On randomized matching mechanisms. Econ. Theory.

[15] Chen J., Niedermeier R., Skowron P. Stable marriage with multi-modal preferences. Proceedings of the 2018 ACM Conference on Economics and Computation.

[16] Aziz H., Biró P., Gaspers S., De Haan R., Mattei N., Rastegari B. (2016). Stable matching with uncertain linear preferences. International Symposium on Algorithmic Game Theory.

